# A pathological fracture and a solitary mass in the right clavicle: an unusual first presentation of HCC and the role of immunohistochemistry

**DOI:** 10.1186/1477-7819-10-50

**Published:** 2012-03-08

**Authors:** Eleftherios I Mantonakis, Theodora S Margariti, Athanasios S Petrou, Anastasios C Stofas, Andreas C Lazaris, Alexandros E Papalampros, Demetrios N Moris, Panagiotis O Michail

**Affiliations:** 1 1st Department of Surgery, Laiko General Hospital, National and Kapodistrian University of Athens, 17 St. Thomas Str. Athens 115 27, Greece; 2 The Oxford Upper GI and Transplant Center, The Churchill Hospital, NHS Trust/Oxford University, OX3 7LI, Oxford, UK; 3 1st Department of Pathology, School of Medicine, National and Kapodistrian University of Athens, Greece

**Keywords:** HCC, pathological fracture, unusual site, clavicle, extrahepatic, immunochemistry, Hep-Par-1

## Abstract

**Absrtract:**

Hepatocellular carcinoma (HCC) is an aggressive malignant tumor that occurs throughout the world. Μetastases from hepatocellular carcinoma (HCC) were generally considered to be rare in the past, because the carcinoma had an aggressive clinical course. In our era, has been reported that extra-hepatic metastases occur in 13.5%-41.7% of HCC patients and this is considered as terminal-stage cancer. The prognosis for patients at this stage continues to be poor due to limited effective treatment. The common sites of extrahepatic metastases in patients with HCC are the lungs, regional lymph nodes, kidney, bone marrow and adrenals. We present here an extremely infrequent case of a patient, without known liver disease, in which the presenting symptom was a pathological-in retrospect-fracture of his right clavicle which wasn't properly evaluated, until he presented a bulky mass in the region 6 months later. For our patient, the added diagnostic difficulty alongside the unknown liver disease, has been that the clavicular metastases was the first presentation of any metastatic disease, rather than the more common sites of HCC spread to adjacent lung or lymph nodes.

## Background

We present here a case of a patient, without known liver disease, in which the presenting symptom was a fracture of his right clavicle which wasn't properly evaluated, until he presented a bulky mass in the region 6 months later. With the help of imaging techniques, tumor markers and immunohistochemistry, we were able to identify the primary site, a tumor in segment IV of the liver and refer the patient for the proper treatment.

Bone metastasis of HCC is rare and usually located in the spine, the pelvic bones and the ribs. A solitary bone metastasis and a pathological fracture in the upper limb, as presenting symptoms of HCC are extremely rare, with only a few references in the literature.

## Case presentation

**Figure 1:**
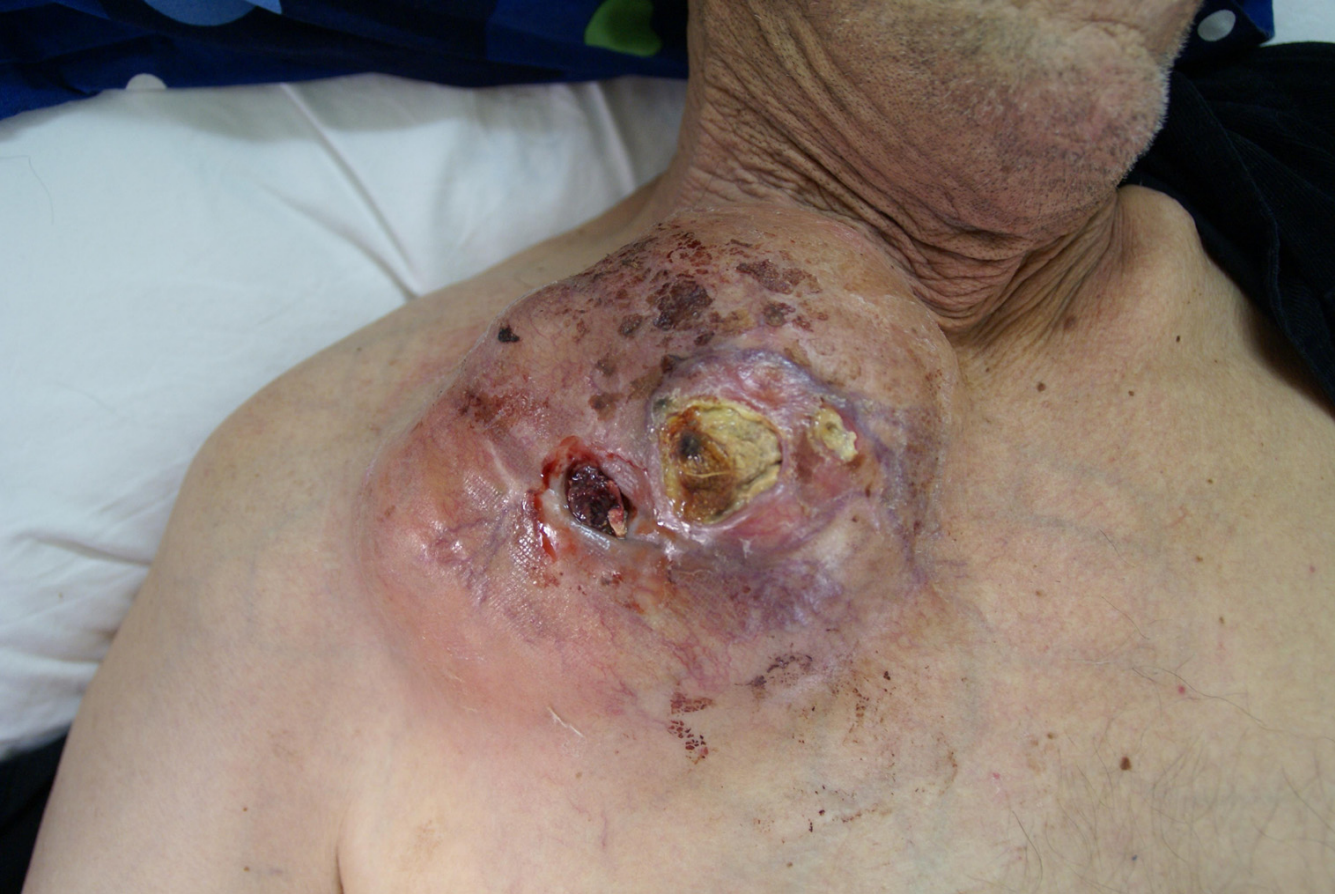
**Patient photo**. Patient photo showing the external bulky right clavicle mass.

**Figure 2:**
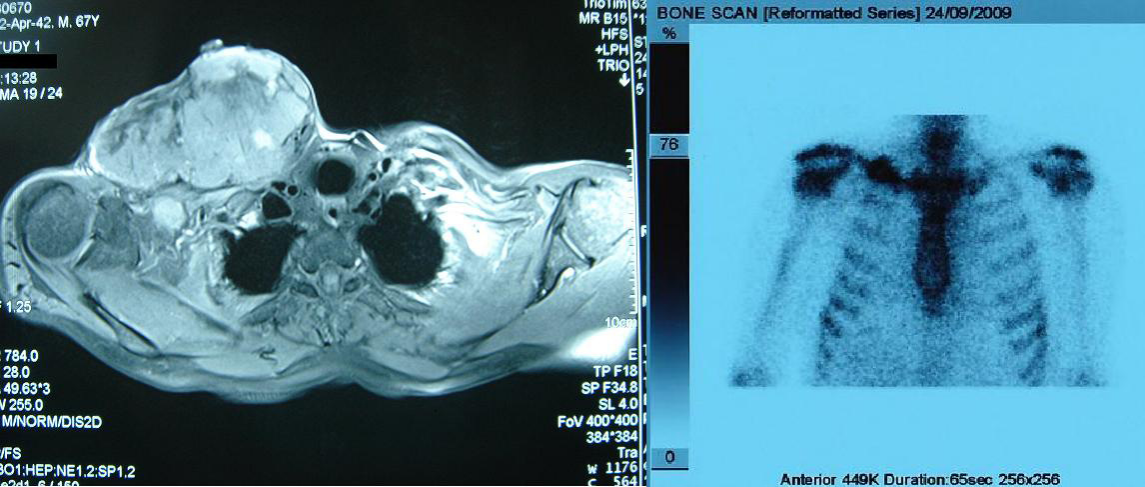
**MRI and Tc 99 m bone scan**. MRI and Tc 99 m bone scan revealing tumor location and relation with the right clavicle.

**Figure 3:**
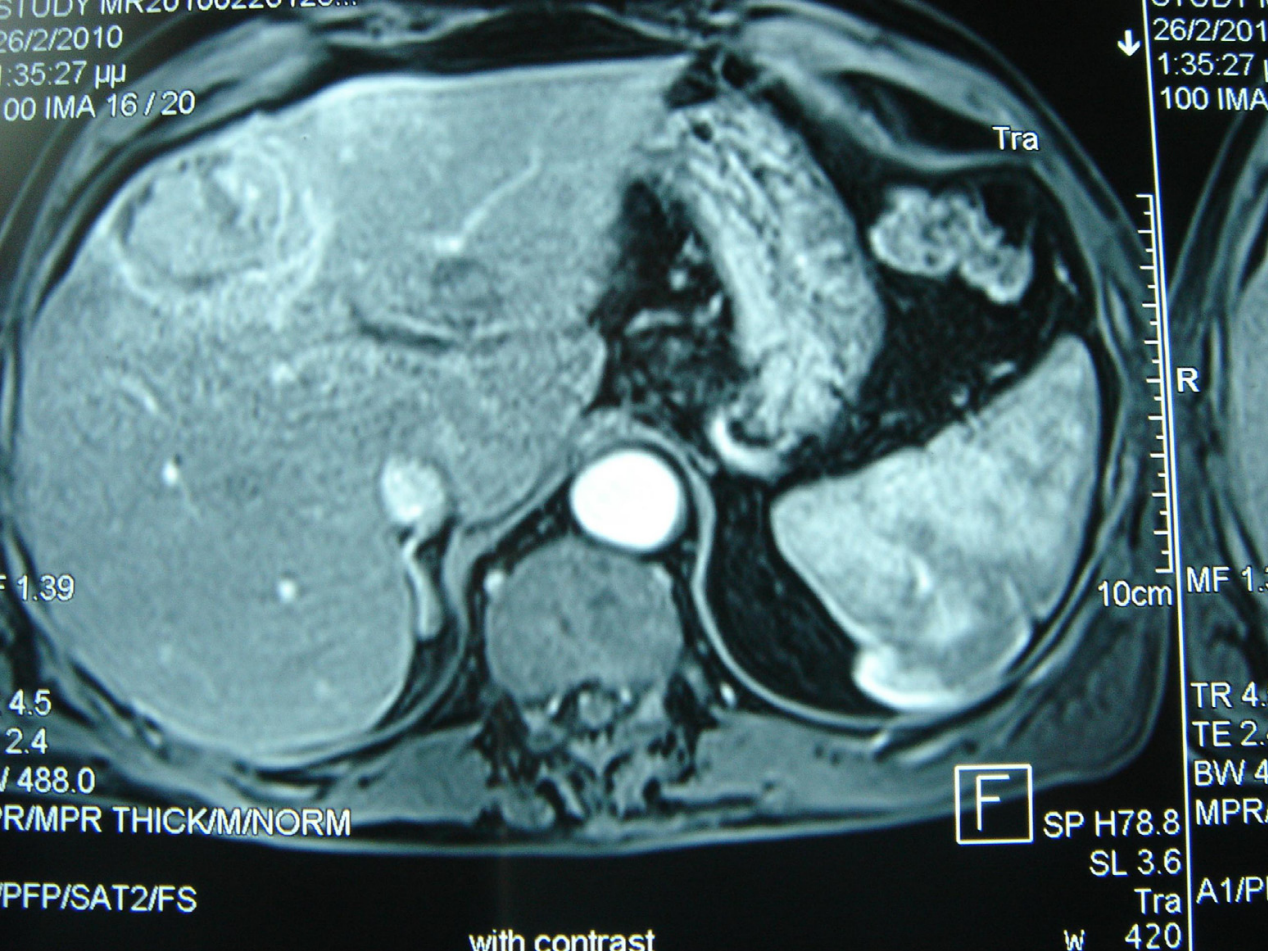
**Abdominal MRI scan**. Abdominal MRI scan showing the tumor at segment IV of the liver.

**Figure 4:**
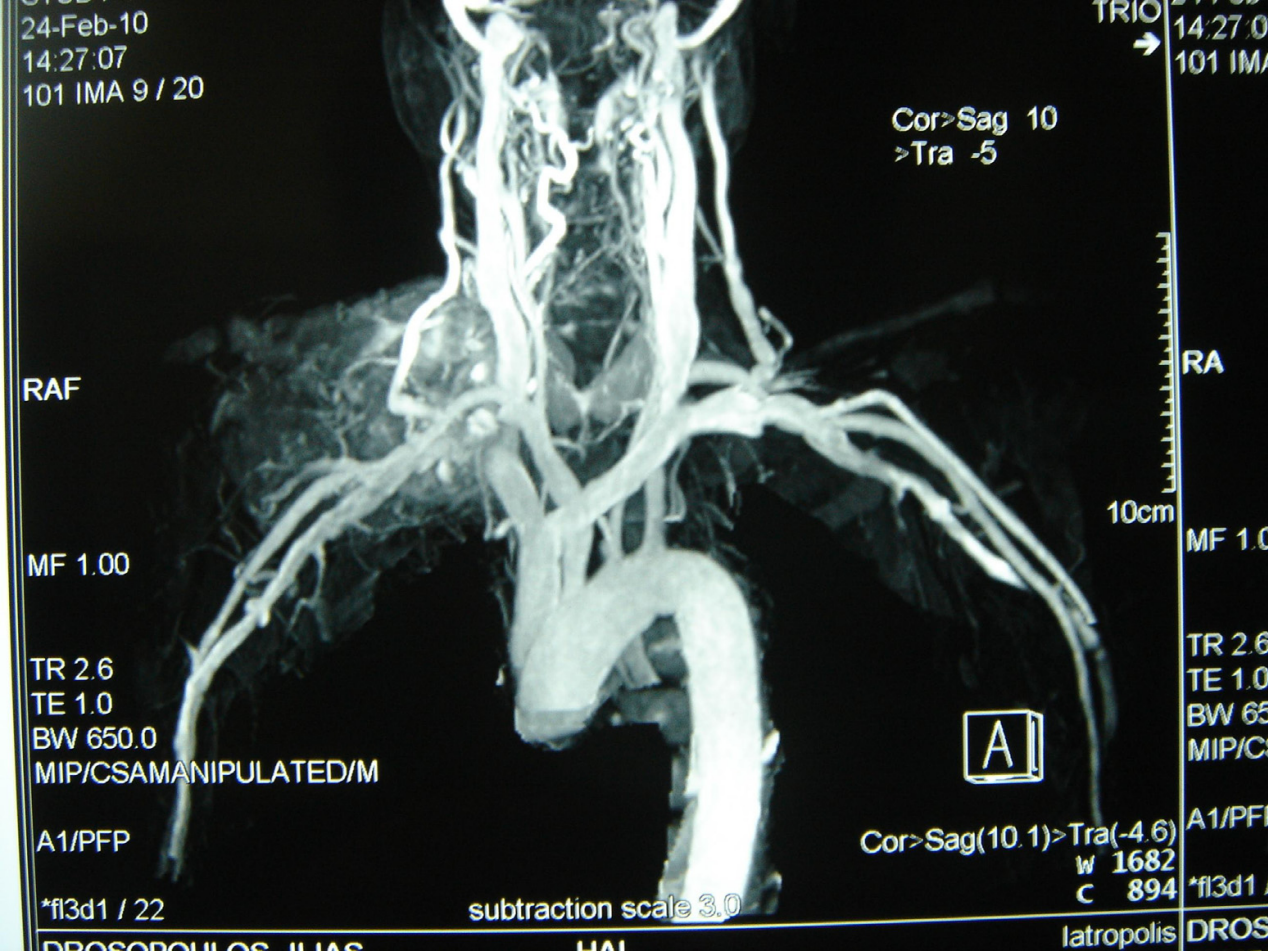
**MRA of the aortic arch**. MRA of the aortic arch showing the relation of the tumor with the junction of the right subclavian vein and the right jugular vein.

A 67-year-old male, with history of a fracture of the medial to inner third of his right clavicle 6 months ago, presented with an impressively bulky tumor in the right clavicle region (Figure 1). From his medical history, a visit in a regional hospital 6 months ago was referred, due to constant pain in his right clavicle. The diagnosis set at that time was a clavicle fracture due to physical activity (farming). He was discharged from the hospital being given conservative treatment. In our hospital, he was subjected as an external patient to an ultrasound of the aforementioned tumor which showed that it was solid and in routine blood tests, which revealed a mild anemia (Hct: 38%, Hb: 12,6 mg/dl) and a normal platelet count (281 K/μl with normal range 140-440 K/μl). We then performed an open biopsy in the Operating Room under local anesthesia in order to identify the type and origin of the mass. Early histopathology exam was positive for malignancy but unable to provide accurate information about the tumor, so the patient was admitted in our hospital for further examination. Blood biochemical analysis revealed elevated liver function tests (AST = 127 U/L, ALT = 94 U/L with normal ranges 5-40 U/L), while the patient's prothrombin time was within normal range (PT = 11,4, INR = 1,01 with normal ranges 10,6-12,6 and 0,85-1,15 respectively). A thoracic CT and MRI followed which revealed that the tumor was surrounding and invading the right clavicle, while smaller lesions with the same characteristics were present at the acromioclavicular junction. A Tc 99 m bone scan showed increased deposition of the drug and disordered bone structure in the right clavicle without any involvement of other bone structures (Figure 2). A colonoscopy and esophagogastroduodenoscopy were performed and the latter revealed a laceration of the larynx near the vocal cords and a possible subglottic mass. The patient was subjected to a biopsy of the area in question which proved negative for any malignancy, drawing our attention away from the neck. Furthermore, he was subjected to an abdominal CT and MRI scan which showed a mass in the segment IV of the liver, with a characteristic contrast solution uptake in the arterial phase possibly representing an HCC (Figure 3). At that time, the histopathological findings and immunohistochemistry were available (see section below) which revealed findings of metastatic HCC and made us concentrate even more on the liver. A sonovue enhanced ultrasound was performed which confirmed our original findings, a 5,7 cm area in segment IV with both central and peripheral uptake on the arterial phase, which infiltrated a branch of the middle hepatic vein, and which was associated with two lesions in proximity that possibly represented regenerative lesions. The patient's blood tests measured by ELISA (MEIA) came positive for HCV, while his tumors markers showed elevated a-FP levels of 18,1 (normal values < 10). As a last exam, we decided to perform an MRA of the aortic arch which showed that the tumor was in close relation with the junction of the right subclavian vein with the right jugular vein, without clear marks of infiltration (Figure 4). The patient was diagnosed with metastatic HCC, due to cirrhosis after HCV infection and was referred to an oncologist in order to receive systemic chemotherapy (cisplatin and epirubicin, along with sorafenib) and local radiotherapy. At that time we characterized, in retrospect, the fracture as "pathological", since the patient never actually mentioned a specific high-energy injury in the clavicle area and the diagnosis previous set was based in the absence of additional evidence.

## Histopathological findings and immunohistochemistry

Grossly, the surgical specimen from the clavicle tumor consists of multiple, gray-white tissue parts of a total volume of 20 cc.

**Figure 5:**
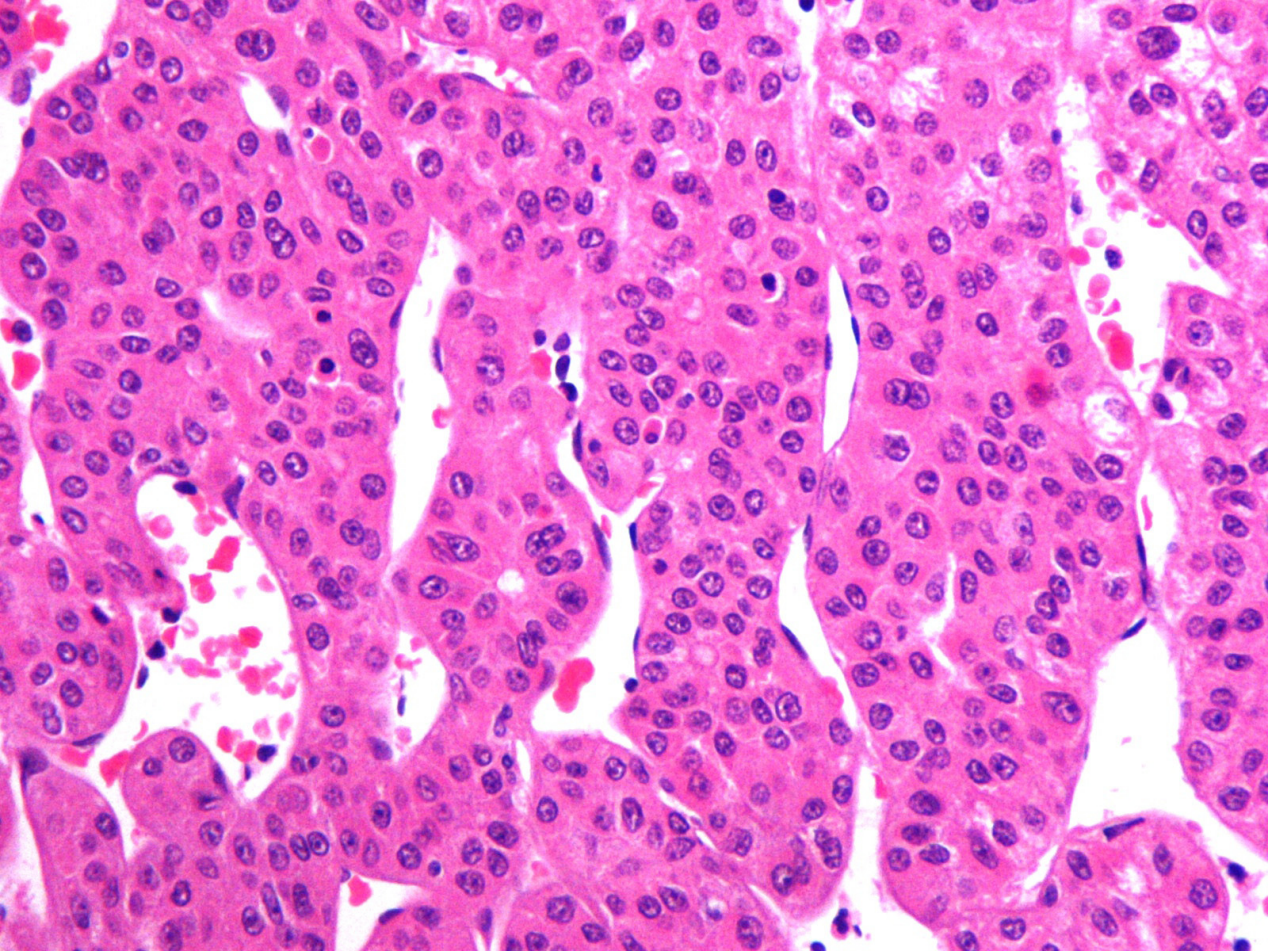
**Hematoxylin and Eosin staining**. Hematoxylin and Eosin staining revealing a neoplasm consisting of cells with eosinophilic cytoplasm, enlarged hyperchromatic nuclei and prominent nucleoli, mostly trabecular pattern of growth and stroma composed of sinusoid-like blood spaces with varying degrees of dilatation, lined by a single layer of endothelial cells.

Histologic examination of the specimen revealed a neoplasm consisting of cells with eosinophilic cytoplasm, enlarged hyperchromatic nuclei and prominent nucleoli. The pattern of growth is mostly trabecular and the stroma is composed of sinusoid-like blood spaces with varying degrees of dilatation, lined by a single layer of endothelial cells (Figure 5).

**Figure 6:**
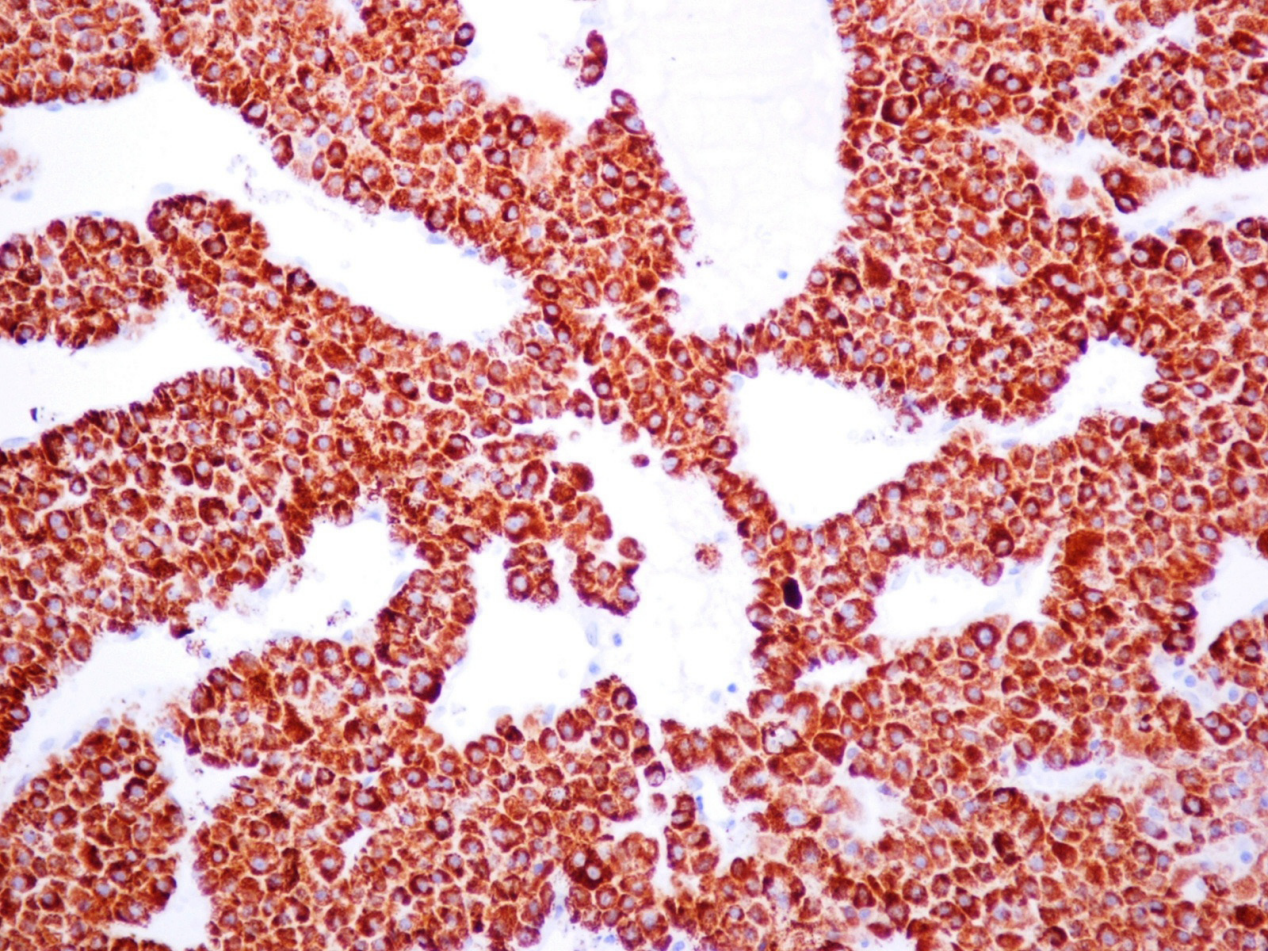
**Immunochemistry**. Immunochemistry with Hep-Par-1 positive staining of the neoplastic cells.

Immunohistochemically, the neoplastic cells show positive staining for Hep-Par-1 (Figure 6), cytokeratin 8/18, CD10 and negative staining for cytokeratin 7, 19, 20, aFP, Ber-EP4, TTF-1, thyroglobulin, vimentin, chromogranin and synaptophysin. According to these, the tumor is classified as a malignant epithelial neoplasm with hepatocellular differentiation, most probably deriving from the liver.

## Discussion

HCC is a malignant tumor derived from hepatocytes. Viral infections (HBV, HCV) and chronic alcohol abuse are the most common etiological factors. Liver cirrhosis is the major clinical HCC risk factor. HCCs, in a percentage of 70-90% develop on the background of macronodular cirrhosis, while males are always more frequently affected than females. A recent cohort study has shown an increase in the number of individuals with HCV in the last decade (from 17,261 in 1996 to 106,242 in 2006). Similarly, the prevalence of cirrhosis increased from 9% to 18.5%, whereas the prevalence of HCC increased approximately 20-fold (0.07% in 1996 to 1.3% in 2006) [1].

The presenting symptoms in patients with HCC include abdominal pain, general malaise, anorexia or weight loss and nausea or vomiting [2]. The majority of patients with extrahepatic metastases experience no specific symptoms, and it is possible to overlook extrahepatic metastases upon examination. Therefore, increased detection of extrahepatic metastases suitable for aggressive treatment may provide great benefit to patients and improve overall survival of those with HCC. HCC metastasis frequently occurs through the intrahepatic blood vessels, lymphatic system or via direct infiltration of the tumor. It has been reported that extrahepatic metastases occur in 13.5%-41.7% of HCC patients which are mostly haematogenous, with the lungs being the most common target, followed by lymph nodes, adrenal glands and bones, especially in skull [3-8]. Hematogenous metastases occur with the involvement of the hepatic or portal veins or the vena cava. The prognosis of patients with HCC is generally very poor. Most studies report a five-year survival rate of less than 5% in symptomatic HCC patients. HCCs are largely resistant to radio- and chemotherapy. Advanced intrahepatic lesions, presence of vascular tumour invasion, elevated tumour markers and presence of viral hepatitis are risk factors for extrahepatic metastasis [9].

Bony metastases of HCC are considered as one of the most frequent extrahepatic metastases sites and occur as multiple metastases in most patients [10]. In a recent study, the outcome of HCC patients with bony metastases was poor [11], which is similar to previous reports [12,13]. Patients with metastases to bone had a median survival of 6.7 months and usually located in the spine, the pelvic bones and the ribs, while localization in the upper and lower limbs is considered rare [14]. In a series of 4953 patients analyzed by Seung Up Kim et al[15] only 37 patients had bone metastasis and only one of them had a clavicle and sternum metastasis at the same time, while there is only a handful of reports with clavicle involvement in the literature and none as it being the first and sole symptom. Our patient prior to his admission was asymptomatic and without known liver disease. The pathological fracture of his right clavicle and the metastatic lesion which manifested right after it were the presenting symptoms. He presented with an impressive solitary tumor in the right clavicle, with rapid growth and an unusual prolonged patient survival which is noteworthy. We need to take into account that he had a pathological fracture 6 months prior to his admission (his liver disease must have been present long before) and that he is still in good condition 1 year after his admission, with the help of systemic chemotherapy and local radiotherapy. There have been reports of solitary bone metastases as presenting symptoms of HCC, in example in the thoracic wall or in the skull, but they are extremely rare and can be found only as case reports [16-18]. It has also been suggested [19] that HCC cases which present with "bone-only" metastasis have better prognosis after hepatectomy and radiotherapy to the bone lesion.

In the diagnosis of the present metastatic tumor, the immunohistochemical marker of Hep-Par-1 was of considerable diagnostic value. Hep-Par-1 is a monoclonal antibody that reacts to an, as yet, unidentified cytoplasmic marker of normal and neoplastic hepatocytes. It may also stain hepatoid adenocarcinomas (HAC) of the gastrointestinal tract [20] which mainly arise in the stomach and sporadically in the esophagus, papilla of Vater, pancreas, gallbladder, large bowel, as well as in several non-gastrointestinal sites, including lung and urinary bladder. Hep-Par-1 may rarely also stain adrenal gland carcinomas and other biliary tract and gastric carcinomas [21]. On a morphologic basis, yolk sac tumors of testis also may display hepatoid phenotype.

As HAC and hepatocellular carcinoma (HCC) cannot be differentiated on the basis of morphology alone, differences in immunohistochemical reaction patterns would be of considerable diagnostic help. HCCs usually stain positively for CK8, CK18 and polyclonal CEA (the latter with a canalicular staining pattern) and are usually negative for Ber-EP4, CK7, CK19 and CK20, as it happens in our case. On the other hand, distinctive features of both primary and metastatic HACs are positive staining for CK19 and CK20. Positivity of virtually all HACs for aFP, CK8, CK18 and the membranous canalicular staining for polyclonal CEA [22], underline their hepatoid nature. In contrast to the above immunophenotype, in our case, CK19, CK20 and aFP are negative.

Gastric as well as gallbladder carcinomas which may stain positive for Hep-Par-1, are usually also positive for Ber-EP4 and CK7 in contrast to HCCs. The latter two markers showed negative staining in our case. Adrenocortical carcinomas are usually positive for synaptophysin and strongly positive for vimentin. Even morphologically, these carcinomas do not show many similarities to our case. Yolk sac tumor may display a hepatoid phenotype only focally though, and in more than half cases, positive staining for aFP is observed [23].

Hepatocellular carcinoma is the tumor which corresponds better to the discussed morphological and immunohistochemical characters of our case. The cells with eosinophilic cytoplasm, the trabecular pattern of growth with sinusoid-like blood spaces lined by a single layer of endothelial cells, as well as the positive canalicular staining for CD10, strongly suggest the diagnosis of a metastatic HCC.

Our case comes as an addition to a few case reports in the literature which describe solitary bone metastasis as presenting symptoms of HCC, with the addition of a pathological fracture which went unnoticed. In this point, the role of the physician in the primary care must be underlined. Each physician must be suspicious to this kind of fractures, where mechanism of injury is not indicative of such a fracture. The role of taking an integrated medical history, seeking for malignant disease is very important, because an unclear history can camouflage the actual cause of the fracture. These fractures are not routinely suspected to be pathological, unless being accompanied by obvious clinical or radiological features of an underlying disease. This can result in a considerable time lag between the first presentation of the clavicular fracture and recognition that it is in fact pathological [24]. Isolated bone metastasis from HCC may not be as uncommon as previously believed. Clinical doctors need to be on alert when evaluating pathological fractures and solitary bone metastasis for the possibility of the liver being the primary site. When biopsy sampling is possible, the use of modern imaging techniques and immunochemistry markers like Hep-Par-1 can prove invaluable in identifying the tumor origin.

## Informed consent

Written informed consent was obtained from the patient for publication of this case report and accompanying images. A copy of the written consent is available for review by the Editor-in-Chief of this journal.

## Competing interests

The authors declare that they have no competing interests.

## Authors' contributions

EIM, TSM and ASP collected and reviewed the data regarding the patient presented and wrote the paper. AEP and DNM were responsible for reviewing the literature and relating its findings with our case. ACS and ACL were responsible for the pathology report and its findings presentation. POM was responsible for reviewing the final format and editing of the article. All authors read and approved the final manuscript.
